# Malingering

**Published:** 1862-10

**Authors:** Tom O. Edwards

**Affiliations:** Chicago


### MALINGERING.

By TOM O. EDWARDS, M. D., Chicago.

The external, invisible signs of internal and invisible diseases, are in many instances embarrassing. The various neuroses have but few symptoms; not referable to the sufferings of the patients, and in most instances we are compelled to rely on verbal communications. In order correctly to treat disease, we must be familiar with its locality and the complaints of suffering organs, as symptoms are sometimes styled; must be observed with care, and interpreted with accuracy. The introduction of physical signs in the treatment of the diseases of the respiratory organs and heart; has measurably removed the doubts and obscurities so long presenting themselves to the profession. We are now enabled to detect the progress and extent of disease in the chest, and correctly to mark its limits and define its character. The phenomena of diseases of the brain and nervous system are more intricate and perplexing; whilst the cavity of the abdomen can with great readiness be explored. Yet, with all our advances in improvement, we are frequently in doubt, not so much so as location; but often as to the *character* of the implication, and the practitioner is frequently compelled to feel his way carefully and await new developments, before a diagnosis can safely be made. No part of the profession is more embarrassing than that which imposes upon a practitioner the decision between *real* and *feigned* diseases. The motives of human action are so various and conflicting, the resorts of the vicious and artful are so numerous as to escape the acumen of ordinary observers, and even sometimes to elude the most astute. Our skill and closest observation will be taxed between real and assumed diseases. The wisest men have been imposed upon, and the most rigid examinations have resulted in false prognosis. Surgeons of the army and navy posts, open to all and alike honorable, often have their qualifications taxed and their judgment tested in the examination of persons in service, whose maladies if real would justify their honorable dismissal and a pension for life. In the every day routine of practice in private life we will need be advised there are assumed diseases; and in the frequency of crime and the present reference of all questions, involving moral torpitude to the medical profession by our courts, we will see the necessity of guarding opinions and correctly basing them, in order to protect individuals and guard the community. To stand, as it were, between the living and the dead; to protect the maniac from the scaffold or to rob it of its just deserts, is both the glory and shame of our profession. The plea of insanity, now so much resorted to, gives us a manifestation not only of the humanity and justice of the times, but indicates a diffusion of knowledge creditable to mankind traceable to our profession. Disdaining the dull scholastic restraints of the ancients, medical writers of the present century, have thrown broadcast before the community, in popular language, facts that have edified and elevated the masses. A spark has flown from our atlas that has illuminated the world, and many causes of human action, on which the mind has heretofore dwelt with horror when comtemplating their results, are now traced to disease; and asylums, instead of prisons; kind medical treatment, instead of scaffolds; are the present curative means for disturbed and diseased moral and mental manifestations. That many are saved whose punishment would have vindicated the justice of severe penalties, none deny; but that any should be punished whose moral condition at the time of the commission of crime rendered them incapable to discriminate between good or evil, all condemn. Better ninety and nine guilty escape, than one diseased, but honest man, suffer. My purpose is to present some of the forms of feigned diseases most commonly found and to give the most reliable means of discrimination. In the army men who feign sickness are called Malingerers; in the the navy, Skulkers; and to such perfection have they brought their science and tact that the oldest army and navy surgeons have been deceived, and dismissions have been granted men whose term of service had not expired, and pensions granted those whose only merit was the possession of a tact sufficient to deceive.

Those who most frequently feign sickness are soldiers or sailors, but Malingering is not entirely confined to that class. The mendicant, to avoid labor; the poor, to obtain public charity, or to impose on private benevolence; some to defraud beneficial societies; others to obtain compensation for pretended injuries, and a large class of persons to avert punishment feign illness. We cannot pass our streets without meeting some person looking for charity; many of these are unfortunate and for these our public institutions have ample accommodations; yet there are others whose lives have been devoted to deception and whose grounds of charity are assumed illness or misspent lives. During the period of the desolating wars of Europe, when that scourge of mankind, Napoleon, carried desolation and terror into every house by his conscription, the art of Malingering was brought to great perfection. Fœdere says "that it was as difficult then to detect a feigned disease as it was to cure a real one." Happily for us no such necessity has existed, and under our wise and just Government no man, except in the preservation of the public peace or the defence of the Republic, can be forced to bear arms. He who enters the army here does so voluntarily and if his courage should fail him or his enthusiasm expire before his time of service, he has the consciousness that three years will soon expire and that subsequent enlistment will be voluntary. To this fact are we indebted to the comparatively few cases of Malingering, when compared with European severity. Two men are now in this city who imposed on the army surgeons in Germany, and obtained discharges in a way amazing and skillful, and whose departure to America followed these discharges so rapidly as to avoid detection. I know an American soldier who was enlisted and discharged three times, during the last war, and who so successfully imposed on the surgeons as to obtain a pension, which he now enjoys. He feigned hæmorrhage from the lungs, by wounding his throat and gums.

As we have before stated, the motives for feigning sickness are numerous. The soldier, to avoid a battle or duty; the hope of gain induces others; and many cases of suits for malpractice on this ground. The records of courts show many cases against surgeons and physicians for the mismanagement of surgical and other cases, and a few are assumed for the purpose of extortion. There are others who deserve our sympathy, who, in order to elicit kindness and attention, feign illness, and by long confinement and habit become ill.

Dr. Copeland, in his dictionary, has classified the various manifestations of these deceptions, and has thus rendered them appreciable and readily understood. He gives four classes: 1st. Pretended or simulated—the person being in a state of health; 2nd. Artificially excited—disorder being actually induced; 3d. Exaggerated—in the description and appearance the patient being indisposed; 4th. Artificially and intentionally increased or exaggerated during its course. In these four modes diseases may be said to be feigned or simulated.

Upon a correct opinion of a medical man, mayrest, not only his reputation, but the interests of humanity, and the pride of character which all should possess who are fairly in the profession, should stimulate us in the acquisition of knowledge for the detection of imposture; and as the diseases feigned are almost as numerous as the real and substantial suffering "to which flesh is heir," we must commence in a classified list and thus relieve ourselves from the embarrassment incidental to detail. The most satisfactory classification of assumed diseases is, 1st, Those obvious to the senses; 2nd, Diseases and defects of a simple kind, not obvious to the senses, but depending upon the description of the impostor; 3d, Those of a more complicated character, consisting of groups of symptoms. It is not my purpose to enumerate all the assumed diseases. This would be but a recital of all known affections—the most commonly resorted to, and those which we most frequently meet in the Army and Navy are increased or diminished size of parts, malformations, ulcers, wounds and superficial inflammations, paralytic affections, spasmodic affections as hysteria and epilepsy.

Travelers in Europe meet in every street in cities mendicants soliciting alms. The humanity of strangers affords a revenue for the support of innumerable lazaroni and street beggars. The Americans are said to be the most liberal in their bounty, and not being accustomed to meet in their walks here such objects of disease, are readily imposed upon. The students and practitioners of medicine in visiting Europe, find almost as many cases of interest in the streets as in the hospitals, and are surprised when informed that a large proportion of them are fictitious or assumed. The most common mode of imposition is the formation of a *tumor* by artificial means. Nothing strikes the passer-by with more sympathy than a large pendulous tumor, and nothing is more readily made. Air can be passed by puncturing the skin, and by a small blow pipe the cellular tissue is inflated. Confine this in proper bounds, the air will remain until absorbed or eliminated through the skin. The butchers in our markets resort to this. The melinguer also resorts to this process to produce ascetis or hydrocele, or hydrocephalus, also similated hernia. It is no easy matter, without carefully handling the parts, to discriminate between the feigned diseases mentioned and the real. We obtain aid by uncovering the tumor, and from its inelastic and soft feeling can adjudge the difference. An infallible sign of imposition is the puncture contiguous, and in every case covered by *adhesive plaster*. The transmissibility of lighter genuine hydrocele would also exist in these cases. The gravity af the tumor would aid the diagnosis, but a puncture carefully covered by an adhesive plaster, would be the best additional testimony of the imposture. A case of assumed hydrocephalus was recently detailed to me by a surgeon of Cincinnati. The mother was a German, and desired to impose on a public charity. She was successful, and the child recovering so rapidly from a disease which ordinarily terminates in death, or under the most skillful treatment is protracted, induced an examination and the puncture was found. His mortification at the deception was very great. The foreign army journals are filled with deceptions not found in private life, such as swallowing quantities of chalk and vinegar, to eliminate the carbonic acid gas, producing tympanitis and dropsies of the upper and lower extremities; are produced by a ligature, or hanging the arm or leg over the back of a chair previously to the hour of the expected visit from the surgeon. The marks of the ligature and pressure are the best means of detecting the fraud. The tympanitis artificially induced is easily removed by numerous doses of glauber salts and tobacco. The patient seldom repeats the fraud after this drenching. Swelling of the joints is induced by rubbing the skin with acrid plants, as the ranunculus acris. This merely thickens the skin and the thickened condition will detect the imposture.

Dr. Hector Gavin, in a work on feigned diseases, gives many amusing cases of frauds, such as the attempted production of a tumor in the abdomen by forcibly elevating the spine whilst lying on the back; the production of a polypus in the nose by the use of the testes of a cock, or the kidney of a rabbit impregnated with fœtid juices. Prolapsus ani has been imitated by the gut of an ox or sheep, or by the everted annal extremity of a cat or hog; and cancer, in one case, by the use of the spleen of a cow and a sponge under the arm pit, moistened with milk; and it is not uncommon for females to practice the deception of simulating pregnancy by the use of a pad. This scheme is sometimes successful on the parties on whom the fraud is practised, rendered susceptible by the consciousness of guilt. The eye and touch will most readily expose the above named deception.

Feigned diseases of the joints are not so readily detected. *Anchylosis* is occasonally resorted to, in order to recover from physicians, in suits for malpractice, or to obtain release from the army. A case of this description came under my observation during the session of the 30th Congress. Hon. Charles Brown, the representative from Philadelphia, asked me to examine a German, who was importuning him for redress against the decision of a court martial. The soldier told a long and plaintive story of a series of outrages perpetrated upon him. His head had been shaved, he stood guard four hours a day for four days; three months pay was withheld, and he was drummed out of camp, on a charge and conviction of malingering, or feigning an anchylosed knee. He came to Washington to obtain from the Surgeon General a re-examination when that was denied, he presented himself before the Committee on Invalid Pensions. A portion of the committee, touched by the man's sufferings and condition, was disposed to report a bill in his favor; the remainder desired further examination, and I examined the man. His countenance indicated suffering, and he had so schooled himself as to cry almost equal to nature. The limb was equal in length with its fellow; but the thigh of the injured limb was one inch and a quarter less, and the calf one inch less in diameter than the healthy limb. It was permanently extended, and resisted all the force I was able or willing to employ in flexing it. On visiting the war department, Dr. Lawson, Surgeon General, put into my hands the papers and testimony of the trial. The man belonged to Capt. Sherman's company of flying artillery; had been in service two years; bore a good character, and was an efficient soldier. He alledged, about one week before the battle of Buena Vista, that while on parade, he was kicked by a horse one inch below the patella, and presenting himself at the hospital, was admitted. The testimony of four surgeons assert, that on his admission, they could discover neither dislocation, nor wound of the skin in or near the seat of the injury; nor was there either heat swelling or redness in the knee-joint. He was ordered to bed, and linaments directed to relieve pain complained of in the joint. The stirring events of the times, the battle so famous in the history of the last war, and the numerous engagements with the sick and wounded, diverted the attention of the Surgeons from the case for more than a month; the man remaining all this time in hospital. Subsequently, means were used to restore the alledged loss of motion in the joint. Blisters, iodine ointment, and frictions were resorted to without avail, and the soldier became importunate for a discharge and a *pension*. These means occupied about four months, and his condition was not improved. One of the Surgeons had occasion to visit the hospital late at night, and was surprised to find the limb *flexed*. He communicated this fact to his colleagues, and a variety of tests were then instituted, which resulted in a complaint of malingering, a trial by court martial, and the punishment already stated. It was ascertained that he had served in Europe; but no fact further than the service was elicited. The means resorted to by the Surgeons to detect the fraud were ingenious. A double inclined plane, similar to Bell's fracture box, was firmly bandaged on the thigh, a pillow placed under it, but no support to the leg, and the joint of the instrument at the knee was loose. During the hours of wakefulness, or the presence of the Surgeons, the patient kept the limb extended; but the watches frequently found him resting the heel on the bed, and during sleep *invariably* found the limb flexed. When aroused the soldier would instantly restore it to the extended position and the power of four men could not flex it. The facts were clearly proved and the court directed the sentence already named, and it was fully executed. The Surgeon General alledged he had no authority to revoke the sentence, and the Committee of Congress would not act unless there was some testimony, fully proving a mistake. On my way from the War Department, and revolving this matter in my mind, I met Dr. W. T. G. Morton, of Boston, so famous as the discoverer of the anesthetic property of Ether, and enquired of him if he had ever applied or heard of the use of Ether in detecting a supposed Malingering. He had not used it, but did not doubt, if there was deception, it could be readily detected by these means. I wrote Dr. Lawson and Mr. Brown, and desired the man to submit to the inhalation, and that I would be present with Dr. Morton to witness the exhibition. The soldier was advised, but refused to submit. The case was dropped in committee, and he was denied access to the Surgeon General for a week, when he agreed to submit. Dr. Morton, with his usual success, induced the full impression of the Ether, notwithstanding the soldier's utmost efforts at resistance. When fully under the influence of Ether, the limb was easily flexed, and the fraud most fully proved in the presence of twenty intelligent surgeons and others. The consternation and mortification of the German, on awakening, to find his limb flexed and the failure of a system of fraud and deception, practiced for more than two years, can be imagined, but not described. This is the first case of the kind in which anasthetic agents were applied, and its complete success renders them our most efficient agents.

In the 3rd Regt., Iowa vols., a private, from a fracture of the patella, (incautiously prognosed by the surgeon,) that he would never be fit for service, attempted for a month the same fraud and was detected by the splint on the thigh, similar to the means described in the foregoing case. He was restored to the ranks and his anchylosis vanished upon a threat of a court martial. In every case of malpractice in which contracted or stiffened joints are the causes of the suits, the use of Chloroform or Ether will most effectually aid the diagnosis.

Curvatures of the spine are sometimes feigned. We always find in these cases the curvature in the dorso-lumbar region, the curve always single; the convex side free from tumefaction or swelling; two folds of the skin on the concave side and the hip raised, and the leg of that side shortened. In diseased conditions there is more than *one* curvature; the convex side is tumefied or gibbous; the seat of curvature variable; there are *no folds* of the skin on the concave side; the trunk has little or no inclination, and but slight elevation of the haunch. In cases of this kind the Ether or Chloroform would unmask the deception fully, independent of the distinct differences pointed out. By long continued bandages and flexion, contraction of the neck, shoulders, limbs and joints have been induced or feigned. These contractives are generally attributed to injuries such as wounds or burns. The absence of scars would create suspicion, and the fullness and firmness of the contracted muscles would confirm it. The means most useful in detection would be a tournaquet on the nerves supplying the contracted limb, applying wet bandages tightly around the limb, which, on drying, so press the muscles as to prevent the contraction; giving emetics or intoxicating, and during the consequent relaxation, moving the limb; suddenly moving the limb when the attention of the patient was engaged, or by applying weights or pullies. The latter expedient is dangerous, as adhesions may have taken place and their rupture may be injurious. The will has been schooled and the muscles trained by use; to bear immense weights; by etherization we overcome the resistance of the muscles and suspend the will. The actual cautery is frequently resorted to to cure real *Coxalgia*, and several cases are reported of *instantaneous* cure. These are feigned, and the patients, dreading a repitition, have ceased their imposition. A constant patient in Commercial Hospital, an Irishman, after spending the summer in a Cholera Hospital, desired a winter's residence for Lumbago. His general health was good. After free scurification and cups, the red-hot iron was ordered in two days if not better. He left before the time, and has never returned, alleging "he was not going to be burnt for a pain in his back."

*Dislocations* are sometimes feigned. I know an individual who can subluxatê his shoulder-joint at will. It resulted from a full luxuration and a laceration of the ligaments. He is something of a wag and has tested the surgical skill of a number of physicians in its reduction and has accustomed himself to bear much traction, and after an acknowledged failure on the part of the surgeon, mortifies and surprizes him by reducing it himself.

Wounds, ulcers, and inflammations of various kinds, are similated by soldiers and paupers. They are difficult to discriminate from the genuine diseases. In countries requiring long and severe military service, such as in Egypt, it is said by travelers, it is difficult to meet with a grown man unmutilated, or feignedly so. Ulcers are frequently artificially induced, and when thus are kept up by irritants. Many patients in our benevolent institutions prefer a residence there to the precarious subsistance of ill-directed labor, or lives of indolence; and thwart the labors of surgeons by use of irritants, or violate directions by assuming positions injurious to their wounds. In these cases, confinement to bed by ordering the nurse to lock up their clothes, and having an attendant to dress the wounds, will frequently expose the fraud; or confining the limb in a tight box, inaccessible to his arts, will surely cure.

Army Surgeons meet a variety of cutaneous diseases similated by soldiers, such as lupus, by pounded garlic or euphorbia; erysipelas, by the short application of blisters, and small pox in its eruptive stages, by punctures, into which bay salt and gunpowder are rubbed. These impositions seldom occur in private practice. Persons from habit acquire the power to vomit at will, and it is a fictitious symtom in disease. It can be induced by swallowing air and by a strong and sudden effort of the abdominal muscles; no inflammation following, and no concomitant symptoms would justify investigation for fraud. I knew a female who, either from habit or the use of nauseants, acquired the power to vomit at pleasure, and was several times ill from inflammation of the stomach thus induced. My candor in informing the husband of the origin of the malady, lost me the practice of the family. The habit was continued until thickening of the mucous membrane resulted, and before her death she confessed the fraud. It was to receive the sympathy of friends and to avoid labor. As we have before observed, almost all diseases are assumed, but those which most decidedly impress the friends or excite most fully the sympathy of physicians, are generally selected. *Hœmoptesis* is a favorite. This is produced by incising or puncturing the gums and throat, or by retaining in the mouth various lozenges, colored with carmine or vermillion. *Hœmaturia* is similated by swallowing beets-madder or cochneal and blood injected into the bladder or obtained from the urethra by wounding and various alledged urinary concretions, found to consist of sand, quartz, flint and glass, have been seen to pass with the urine. One of the most remarkable of these cases is recorded in the *American Journal of Medical Science*, 14th vol., page 91, by Dr. Caleb Ticknor: Miss P., of Massachusetts, had been bed ridden for some twenty years, in consequence of an accident received when eleven years of age, by a barrel falling across her loins. She suffered for a considerable time pain in various parts of the body, as the loins, stomach and bowels, and finally lost the use of her limbs. When Dr. Ticknor was first called in consultation she was laboring under a severe diarrhœa, and pretended that food taken would pass the bowels in ten minutes after swallowing, without having undergone any change in smell or color. From this time she pretended to have gone one hundred and nine days without any evacuation from the bowels. After this she is reported to have vomited the contents of an enema in a few minutes after it was administered, without any admixture of fœces. During the one hundred and nine days, she is said to have vomited regularly every day, the food, properly digested, which she had taken during the preceeding twenty-four hours, and with this, as well as the urine, *sand* was intermized. The bowels now resumed their office and the urine was vomited. On trying to pass a catheter a hard substance was found in urethra, blocking up the passage. Occasionally, it is said, the urine was passed per rectum, mingled with sand, and at length after the urine began to flow through the natural passage lumps of concrete sand were discharged with it. Finally an abscess is said to have formed in the side, from which escaped sand, pus and fœces. These phenomena are said to have recurred for several weeks together, until at length the patient insisted she had no fœcal or other evacuation from the stomach for forty days, except urine, while she occasionally vomited, together with injections, fifteen minutes after they had been administered, but without any appearance of fœces. The tongue was black as ink, except the edges, which were red. She complained of excessive pain in the right side and stomach; said she could feel lumps of sand moving about inside; sand continued to pass from the side, mixed with blood and fœces and sometimes milk, and she pretended to have spasms about the throat and mouth. Dr. T. states he could not succeed in passing a probe into the orifice of the abscess, but he could feel the sand inside. One day she pretended to have passed from the bowels forty-four lumps of sand, at one sitting, varying in size from a pea to a walnut, and vomiting of fœces and urine seems to have been a common occurrence for months together, although her jaws were firmly set. Blood is also said to have flown from her mouth, ears and nose, commingled freely with sand, and her mouth is said to have been filled with sand for eleven consecutive days. Dr. Ticknor says he saw her vomit a gill of urine, perfectly transparent, and also a teaspoonful of fluid discharged from the ear with sand, and a lump of sand from the nose. On the 1st of August, he states, she voided by stool a tablespoonful of sand with a tin cup full of pus; also a membrane-like substance of the size of a crown piece containing *fine delicate hairs*, together with sand and urine from the mouth, rectum, urethra, nose, ear, side umbillicus. A month after this we find her still vomiting urine and affected with spasms, resembling epilepsy, with the left leg strongly flexed upon the thigh, and the heel drawn up and resting on the gluteii muscles for twelve days together. From the 20th of July to the 20th of September, she claimed to have no natural evacuation from the bowels or bladder, but had vomited the contents of both. A year after, Dr. T., on visiting her, received the following account from her sister: "That her bowels become regular soon after his last visit and continued so for some time, when she again vomited their contents; appetite generally good; left leg during the whole time continued flexed, and attempts to straighten it occasioned frightful spasms; had rode out frequently; had done a good deal of needle work, and enjoyed comparatively good health." A portion of the sand was analyzed and was found to be composed of silix and lime, mixed with a few short hairs, precisely resembling the mortar from a dwelling. I consider this one of the most remarkable instances on record, as the patient continued for many months to impose on all the physicians who visited her, amounting to at least a dozen. In commenting on the case, Dr. T. admits that she might have practiced a deception, but that such a supposition was hardly probable, inasmuch as she sustained a character for genuine piety, and as she and her two maiden sisters enjoyed a competency, there was no motive for them to deceive. The Dr., at the close of his narrative, says, "the patient is living about fifteen miles from where my brother is now practicing, and he may yet detect her in her imposture, if she is not put upon her guard by publishing speculations prematurely." That a number of physicians were imposed upon by this woman is apparent from the belief in the occurrence of these phenomena. A bare recital of the facts would be sufficient to convince the most casual observer of the fraud practiced. The sisters were accessory, for to believe that this fraud could exist for so long a period without their cognizance, is absurd, and beside *their statements* are mostly received as proof the occurrence. The glass and silex passing her bowels would have been sufficient to detect her. The statements of the Dr. in relation to the circumstances of the family were found to be erroneous, and gain was the inducement. A similar instance occurred in Maryland, in which there was no pecuniary necessity or desire, but can only be accounted for on the ground of moral insanity.

The conduct of those alledging themselves to be under the influence of witchcraft, as exhibited in the early settlement of this country, are examples of feigned sickness, and may be traced to every motive that could possibly operate upon mankind. Fear, malice, gain, and moral insanity, will account in every case, for the outrages there committed by witnesses, and these can only account for the most singular conduct of persons whose intelligence and position would indicate a better state of affairs. Bancroft gives many scenes in his history of these times, and were it not that the mind is horrified with the injustice done innocent persons, and staggered at the credulity of men of acknowledged character and intelligence, they would afford a cabinet of Momus. The bewitched person in one case pretended to be dumb. "Who hinders these persons from giving testimony?" "I suppose the devil," answered the accused. "How comes the devil," retorted the Chief Judge, "so loathe to have any testimony brought against you ?"—a rejoinder pointed, and looking with great power to the supposed facts of the case.

No man in society but that may innocently become the subject of malicious prosecution. A clergyman, hearing his wife and domestic disputing in the kitchen, about some trivial matter, went down and only interfered so far as to repel some rudeness offered by the girl to her mistress, by pushing the girl one side. She either accidentally or purposely fell against the dresser and received a slight wound over the eye, of no sort of consequence. She immediately went up stairs, stood at the street door, told the people passing by; that she had almost been murdered by her master, and to confirm this statement dropped into an apparent epileptic fit. She was then taken as one expiring to a hospital, and without further enquiry the unfortunate clergyman and wife were dragged to Newgate. The indignation of the populace was so great that the windows were broken and the furniture in his house was mutilated and thrown into the streets. In the evening the account of this dreadful murder was cried through the streets by the newsboys. The next day the patient was removed from the hospital to private quarters, where she was attended by the faculty for ten or twelve days, during which time she never exhibited the least sign of sense or memory, but seemed to be (particularly when examined) violently agitated and convulsed. Prof. Sam'l Cooper was called in consultation, and on examining her carefully was rather surprized to find the case one of Malingering. In this connection one of two purposes presents itself: either to alarm the patient by the fear of an operation, or to arouse her into a sudden passion by abuse. The Dr. took the latter method, threatened to send her to prison for the imposture. She become as one "electrified, bounded from her coma, and *swore he dare not.*" The girl immediately decamped, the clergyman and wife were released, and the physicians in attendance were taught a lesson in *diagnosis* that lasted them to their last moments. The terror and shame of the clergyman was so great as to jeopard his life, and he recovered after a long illness with impaired health, standing and fortune.

A gentleman of my acquaintance, of some celebrity as a surgeon, was called to see a negro man, found in a barn on Sunday night, bleeding from the nose and mouth, and in most perfect coma. The signs of cerebral pressure were very manifest, and a bruise on the head seemed to indicate fracture with depression. The head was carefully shavred and the trephine prepared for the elevation of the supposed depressed bone. The incision was made through the tumefied scalp and the bone exposed, when a student present thought he perceived the odor of rum in the breath of the patient. The stimulus of the knife had somewhat aroused him and a few words in an under tone obtained the confession of facts that suspended any further progress in the operation. The head was carefully bandaged; cold cloths applied; a fee of fifty dollars charged for the operation—the master of the slave to this day in blissful ignorance of the facts of the case, and the surgeon added an additional leaf to the chaplet, encircling his brow, *i. e.*, one in the estimation of the public. His student, one or two medical friends and himself are the only parties cognizant of the affair, and each of them have added a note in his case book "never to prepare for or begin an operation for injury of the head without previously smelling the patient's breath."

*Epilepsy* is frequently feigned both in and out of the army, and so artful and persistent are the means employed, as to render the fraud most difficult of detection. No disease in the vast vocabulary has received more medical investigation; none so fully challenges public sympathy. All admit its distinctiveness and mourn the impossibility of relief in many cases. No sensible man now gives the interpretation from its name as a visitation from above. It is assumed, because there are intervals of apparent health in the real disease, and the imposter can suit his convenience in inducing the fit. The real epileptic is oftentimes bruised and injured during the convulsion; the feigned resorts to similar means to confirm the impression he desires to make. One sign of the imposition is the frequent recurrence of the patient to his condition—the warning of those around him of the attack, and his constantly making his infirmity the ground of favor or sympathy. The real epileptic will never, unless interrogated, speak of his affliction, and if intelligence be not destroyed; will studiously avoid any conversation upon the subject. Besides, the epileptic is never importunate for medical assistance; the feigned a constant attendant upon physicians, whose money, not remedies, he mostly covets. During epileptic seizure, there is an entire loss of sensibility; the most intense stimulants, such as fire or puncture fail to produce any impression. These are cruel means, and would be justified only when there was strong evidence to ground suspicion that the case was a deception. The safest stimulants are the vapors of ammonia, snuff applied to the nostrils, assafœtida, alcohol to the eye. These fail in real epilepsy, and cases are recorded of the insensibility to these impressions being acquired. Fodere relates a case of a soldier who had acquired such skill in feigning, and such insensibility to stimulants, as to resist the application of all but *fire.* This always restored him, though he lay apparently without sense, his eyes starting from their orbits, and his mouth foaming. He afterwards confessed the deception. This was a violent measure, and should never be resorted to, unless we have the absence of two symptoms ever attendant upon epilepsy—the dilatation of the pupils, their entire insensibility to light, and a natural sleep following each paroxysm. We repose more confidence in these two facts than any combination; indeed we regard them as *sine qua non.* If, after epileptic seizure, a patient should immediately arise and walk, and during the convulsion light contracted the pupil, we have just grounds of suspicion and are fully justifiable in the use of harsh means for their confirmation. The biting of the tongue or the expectoration of blood from wounds of the mouth, the convulsive action and frothing at the mouth may be assumed; but a dilatable pupil or the sudden recovery and assuming the erect position, with facile locomotion, are never found in genuine epilepsy. A girl was brought to the hospital at Vienna, who had been cured of deafness, which was speedily followed by epilepsy. The fit which did not occur more than once or twice a day before her admission, now recurred every hour. It resembled a real invasion, by the clenched hands and distorted eyes. The surgeons suspected deception, for the following reasons: she did not open her eyes, during a paroxysm, with a wink, but in the natural way; pulse natural; when the curtains were drawn, the pupils were dilated; on opening the eyes the pupil contracted, and when a candle was presented to the eye, the contraction was violent. This convinced the surgeons the disease was assumed, and ordering her to be taken from bed, and kept in an erect posture, with further instructions if she fell to chastise her severely, brought the confession in a day or two. The pupil in this case gave the diagnosis. Another case of a female, confined to prison for murder, who had resisted three severe burnings and had fully convinced her attendants of the realty of her affliction, but betrayed herself by rising immediately after a fit, and walking steadily. The remedy resorted to in Paris and found frequently successful in epilepsy, is placing the patient upon straw and setting fire to the corners. It is said to have succeeded in the hands of the police in many instances of long practised deception. The diagnosis is important, and we repeat it, if the pupil during a paroxysm is insensible to light, if sonnd sleep follows, and a consciousness of affliction that induces the patient steadily to avoid conversation on the subject, the disease is not assumed. If these are absent, we may with great propriety question the seizure.

*Paralysis*, and consequently loss of motion and sensibility, are frequently feigned. Partial paralysis is always preferred to general, as it is better assumed. A general history of the case or loss of temperature of the affected part in real disease, will give the grounds of difference. If any doubt exists, a careful watch and low diet will in time give developements, besides the diet is one of our best therapeutic agents. *Insensibility*, *coma* and *lethargy*, all similar manifestations of severe diseases, are assumed. That so high a degree of tolerance of pain could be assumed, as we have evidence of, is a phenomena alike curious and inexplicable. The most violent irritants are resisted, and one case is related of the scalping of the head and application of the trephine having failed to extract a sign of consciousness. A soldier by the name of Drake assumed entire insensibility and resisted for months every variety of treatment; even the shower bath and electricity made no impression upon him. A proposal to apply the red hot iron was more than he could contemplate, and before the instrument could be prepared, he decamped. However, Phineas Adams, a soldier of the Somerset Militia, was confined in jail for desertion. From the 26th of April to the 8th of July, he laid in a state of insensibility, resisting every remedy, such as thursting snuff up the nostrils, electric shocks and other potential agents. When his limbs were raised, they fell with the leaden weight of inanition. His eyes were closed and his countenance pale; his respiration free, pulse natural. The sustenance he received during this period was eggs and wine, and occasionally tea, which he sucked through the teeth, as all attempts to open the mouth were fruitless. Pins were thrust under his finger nails, to excite sensation, but in vain. It was conjectured the illness was owing to a fall, and a proposal was made to remove the pericranium to ascertain the existence of the depression of the skull. The surgeon described the operation to his patient, at his bedside, and actually removed the scalp and examined the bone. During the operation, no emotion escaped him, and but once, when the trephine was applied, did he manifest any sensibility, and then only by a groan. The life of the man not having been destroyed, nor his health benefited by this dernier resort, he was discharged and sent home; in two days thereafter, was seen sitting at the door, talking; next day, walked two miles, cut spars, carried reeds up a ladder, and assisted his father in thatching. This is a remarkable case, and one that startles our credulity; yet the attestants are credible. *Hysteria* is occasionally feigned, and so artfully as to be difficult of detection. If an imposture of this disease be treated with the nauseating draughts usually prescribed by the routinist, or an occasional shower-bath of cold water, a remedy equally profitable in the real in many cases, as in the assumed disease, we either cure the affection, or, what is equally beneficial, induce its being no longer practised. *Syncope* is sometimes feigned, but is readily detected. It is not an uncommon occurrence on the part of young ladies of delicate sensibilities, and although those who really faint always fall, yet these take good care to have some one near, into whose arms they may find a lodgme[illegible]. Members of Congress have been known to faint at the conclusion of long speeches. It was a common finale, until the sagacious John Randolph, of Roanoke, spoiled all its artistical effects by observing the singularity of the phenomena, that the face of the fainting member continued *red*. On one occasion he said: "Col. ——— made a good speech, but not so good a faint, as his face was too red." The remark gave rise to lengthy correspondence and a threatened duel, but was the means of breaking up the growing evil.

*Death* has been feigned; in the case of the celebrated Col. Townsend we have a remarkable illustration. We quote a description from Dr. Cheyne: "He told us he had sent for us, to give him some account of an odd sensation he had for some time observed and felt in himself, which was, composing himself, he could die or expire when he pleased, and yet by an effort, or somehow, he could come to life again; which it seems he had sometimes tried before he had sent for us. We all three felt his pulse; it was distinct, though small and thready, and his heart had its usual beatings. He composed himself on his back and lay in a still posture for some time, while I held his right hand; Dr. Baynard laid his hand on his heart, and Mr. Skine held a clean mirror over his mouth.

I found his pulse sinking gradually, till at last I could not feel any, by the most exact and nice touch; Dr. Baynard could not feel the least motion in his heart, nor Mr. Skine discern the least soil of breath on the mirror. Then each of us by turns examined his arm, heart and breath, but could not by the nieest scrutiny discover the least symptom of life in him. This continued about half an hour. As we were going away (thinking him dead) we observed some motion about the body, and upon examination found his pulse gradually returning and motion of his heart; he began to breathe gently and to speak softly. This experiment was made in the morning and he *died* in the evening. On opening the body, nothing was discovered but disease of the kidney, for which he had long been under medical treatment, all the other viscera being perfectly sound. This case is remarkable and instructive; showing states of the system so nearly resembling death as to deceive medical men, and only distinguishable from it by the absence of rigidity and the presence of animal heat. It was a case of voluntary syncope and showed extraordinary influence of the will over the action of the heart. Col. T. is reported even in health to have been enabled to control the action of the heart. I know of no similar case reported.

Should you deem this paper of sufficient importance to give it place in your journal, I will continue the subject under the second head of "Diseases not appreciable to the Senses, but *dependent* upon the description of the Impostor."

